# Serotype Characterization and Transmission Modelling of Foot‐and‐Mouth Disease in Dairy Farms, Bishoftu, Ethiopia

**DOI:** 10.1002/vms3.71054

**Published:** 2026-06-23

**Authors:** Eyerusalem Fetene, Dereje Shegu, Ayelech Muluneh, Abde Aliy, Jan Paeshuyse, Fanos Tadesse Woldemariyam, Haileleul Negussie, Samson Leta

**Affiliations:** ^1^ College of Veterinary Medicine and Agriculture Addis Ababa University Bishoftu Ethiopia; ^2^ Faculty of Bioscience Engineering, Department of Biosystem, Division of Animal Health Engineering, Laboratory for Host‐Pathogen Interaction for Livestock Leuven Belgium; ^3^ Animal Health Institute Sebeta Ethiopia; ^4^ Armauer Hansen Research Institute (AHRI) Addis Ababa Ethiopia

**Keywords:** basic reproductive rate, dairy farms, FMD, SIR model, transmission dynamics, Ethiopia

## Abstract

**Background:**

Foot‐and‐mouth disease (FMD) is among the most important transboundary animal diseases, causing widespread economic losses due to decreased productivity, trade restrictions, and costly disease management efforts.

**Objectives:**

This study aimed to characterize circulating FMDV serotypes and quantify within‐farm transmission dynamics during active outbreaks in Bishoftu, Ethiopia.

**Methods:**

Five epithelial tissue and 39 oral swab samples were collected from 11 dairy farms experiencing active FMD outbreaks. Samples were tested using quantitative reverse transcriptase polymerase chain reaction (RT‐qPCR) and antigen‐capturing enzyme‐linked immunosorbent assay (ELISA) for FMDV detection and serotyping. A susceptible‐infected‐recovered (SIR) model was fitted to outbreak data to quantify transmission dynamics.

**Results:**

Out of 44 samples, 37 (84.1%) were positive for FMDV using RT‐qPCR, and 23 (52.3%) were positive by ELISA. Serotypes O, SAT‐1, and SAT‐2 were identified, with SAT‐2 being predominant. Transmission modelling indicated an average transmission rate (*λ*) of 0.82 and a recovery rate (*γ*) of 0.32 per day, corresponding to a mean basic reproductive number (R_0_) of 3.7. The trivalent vaccine demonstrated low effectiveness (31% against single serotypes; 26% when adjusted for mixed infections), which was insufficient to achieve herd immunity given the high transmission intensity.

**Conclusions:**

These findings demonstrate that vaccination alone cannot control FMD in these systems. Control requires improved vaccine matching and the combination of vaccination with enhanced biosecurity measures, such as animal isolation and movement control.

## Graphical abstract

FMD outbreaks in dairy farms in Bishoftu were characterized by rapid within‐farm transmission (R_0_ = 3.7) and predominance of the SAT‐2 serotype. Current trivalent vaccination alone was insufficient for control, highlighting the need for improved vaccine matching and strengthened biosecurity measures

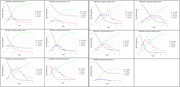

## 1 Introduction

Livestock is considered an integral part of Ethiopian agriculture, which accounts for about 45% of the total value of agricultural production and supports the livelihoods of the largest portion of the population (Wakaso et al. 2025). Dairy production is a high‐value sector that can stimulate agricultural and economic growth while providing valuable income opportunities for the poor (Minten et al. 2020). However, the dairy production is constrained by several factors mainly by infectious diseases. Among these diseases, foot and mouth disease (FMD) ranks among the top five important livestock diseases that have a significant socio‐economic impact on Ethiopia dairy production (Gizaw et al. 2020; W. T. Jemberu et al. 2016).

FMD is a highly contagious transboundary viral disease of cloven‐hoofed animals (Calkins and Scasta 2020). FMD is among the most important transboundary animal diseases, causing widespread economic losses due to decreased productivity, trade restrictions, and costly disease management efforts (Grubman and Baxt 2004; Shapiro et al. 2015). It is caused by a non‐enveloped RNA virus within the family Picornaviridae and genus *Aphthovirus* (Grubman and Baxt 2004; Office International des Épizooties (OIE) 2021). Foot and mouth disease virus (FMDV) comprises seven antigenically distinct serotypes (O, A, C, SAT‐1, SAT‐2, SAT‐3, and Asia‐1), which exhibit limited cross‐protection, necessitating region‐specific vaccine formulations (Ayelet et al. 2009; Davies 2002). In Ethiopia, serotypes O, A, SAT‐1, and SAT‐2 have been documented, with frequent outbreaks causing substantial disruption to livestock production systems (Wubshet et al. 2019).

Bishoftu represents one of Ethiopia's most intensive dairy production hubs, characterized by high cattle population density, a concentration of commercial and smallholder dairy farms, and frequent livestock movements associated with marketing, breeding, and feed acquisition (Megersa et al. 2011). These factors increase the likelihood of direct and indirect animal contact, facilitating rapid FMD transmission within and between herds (Seyoum and Tora 2023). In addition, informal marketing practices, limited biosecurity measures, and frequent introduction of replacement animals from surrounding areas further heighten the risk of viral introduction and persistence (Moje et al. 2023). Consequently, Bishoftu functions as an epidemiological “hotspot” where FMD can spread quickly, evolve through circulating serotypes, and cause repeated outbreaks. A previous study reported a sero‐prevalence of 11% from Bishoftu (Belina et al. 2016). This makes the area particularly suitable for studying within‐farm transmission dynamics, characterizing circulating serotypes, and generating evidence to inform region‐specific control strategies, including targeted vaccination and improved biosecurity.

Mathematical models are powerful tools for understanding the spread of infectious diseases, estimating key epidemiological parameters, and informing the design of effective control strategies (Brauer and Castillo‐Chavez 2012). Among these, compartmental models, such as the susceptible‐infected‐recovered (SIR) model, are widely used in epidemiology to quantify transmission dynamics and predict disease progression (Kermack and McKendrick 1927; Roosa and Chowell 2019). The application of such models to FMD can provide valuable insights into disease spread within herds and inform targeted intervention strategies, such as vaccination or movement restrictions (Bauch et al. 2005; Van Den Driessche and Watmough 2002). In this study, the SIR model was selected because the outbreaks involved acute infections with clearly observable clinical states (susceptible, infected, recovered) and because of the limited availability of detailed latency‐period and animal‐contact data required for more complex models.

Although serotype circulation has been described in Ethiopia, quantitative evidence on within‐farm transmission dynamics remains scarce, particularly in semi‐intensive dairy systems where high animal densities may amplify FMD spread (Tadesse et al. 2019). Therefore, this study aimed to (1) identify circulating FMDV serotypes in Bishoftu dairy farms during active outbreaks and (2) quantify within‐farm transmission dynamics using an SIR model to estimate key epidemiological parameters and inform targeted control measures.

## 2 Materials and Methods

### 2.1 Study Area

**FIGURE 1:**
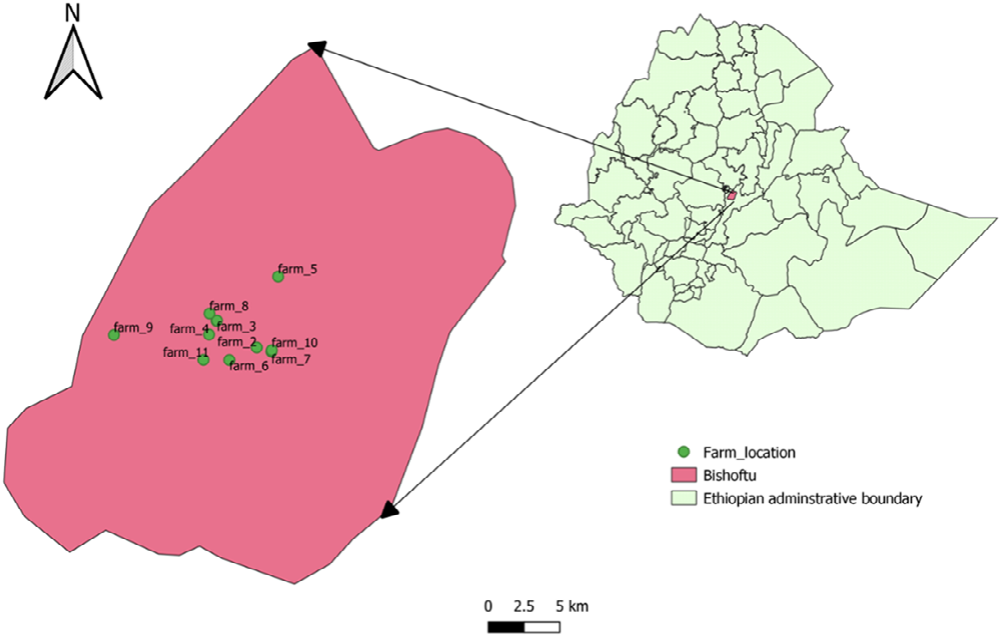
Map showing Bishoftu town and the specific locations of the dairy farms.

This study was conducted in dairy farms located in Bishoftu, Ethiopia, where active outbreaks of FMD were reported between November 2020 and March 2021. Bishoftu is situated in the Eastern Showa zone of the Oromia region, at an altitude of 1920 m above sea level, and coordinates 8°43′–8°45′N latitude and 38°56′–39°01′E longitude (Figure 1). The area is known for its intensive dairy production systems.

### 2.2 Study Population and Design

The study targeted dairy farms experiencing FMD outbreaks. Cattle of varying ages, sexes, and breeds within these farms were included in the study. An outbreak follow‐up investigation was conducted, and farms reporting active cases were visited within 24–72 h. Each farm was considered an independent epidemiological unit, and outbreak monitoring, animal management, and transmission estimation were conducted separately for each farm. Purposive sampling of clinically affected cattle was employed to maximize detection probability during acute infection.

Based on clinical status, cattle were categorized as susceptible (no signs), infected (clinical signs present), or recovered (lesions healed, no new signs for 7 days).

### 2.3 Sampling and Sample Collection

Purposive sampling targeted cattle exhibiting clinical signs consistent with FMD. A total of 44 (39 oral swabs and five epithelial tissue) samples were collected from 11 dairy farms. The epithelial tissue samples were collected from vesicular lesions using sterile plastic tubes containing a viral transport medium (VTM), and the oral swabs were collected from salivating cattle. Swabs were placed in tubes containing 2 mL of VTM, following OIE guidelines (OIE 2021). Samples were transported to the Animal Health Institute virology laboratory using ice packs and stored at −20°C until analysis.

### 2.4 RNA Extraction, FMDV Detection, and Serotyping

Total RNA was extracted from epithelial suspensions and centrifuged swab suspensions by using an RNeasy mini kit (Qiagen, USA) according to the manufacturer's protocols with the final volume of 60 µL RNA, and the extracted RNA was stored at −80°C until further analysis. A probe‐based quantitative reverse transcriptase polymerase chain reaction (RT‐qPCR) assay was conducted to detect FMDV genomic RNA, using previously published primers FMD 3D forward (5'ACT GGG TTT TAC AAA CCT GTGA‐3') and FMD 3D reverse (5'GCG AGT CCT GCC ACG GA‐3') and TaqMan probe (5'‐[6FAM] TCC TTT GCA CGC CGT GGG AC [TAM]‐3′) targeting the 3D(pol) gene. The detailed description of the RT‐qPCR could be found in Callahan et al. (2002). FMDV serotypes were identified using an antigen‐capturing ELISA (IZSLER, Italy), as per the manufacturer's protocol. The ELISA microplates were pre‐coated with capture Monoclonal antibody (MAbs), and positive inactivated controls were incorporated into plates. Epithelial suspensions and centrifuged swab suspensions were incubated with the coated cells, allowing the virus to be captured by the corresponding serotype‐specific MAb and by the pan‐FMDV MAb. The assay is designed to detect and type FMDV serotypes O, A, C, SAT‐1, SAT‐2, and Asia‐1. The IZSLER serotyping ELISA has a reported sensitivity of 79% (Grazioli et al. 2020), while its specificity is not reported in the available literature. Optical density values were measured at 450 nm, and results were interpreted based on the manufacturer's criteria.

### 2.5 Data Preparation for Modelling

For transmission modelling, daily case counts were compiled for each farm from outbreak onset until no new clinical cases were observed. Clinical status classifications (susceptible, infected, recovered) were verified through farm records and follow‐up visits.

### 2.6 Transmission Dynamics Modelling

To model the transmission dynamics of FMDV, a SIR compartmental model was developed using the “DeSolve” package in R (Soetaert et al. 2010). The cattle population within each farm was divided into three compartments: susceptible (S), infected (I), and recovered (R). The following assumptions were made: (1) no birth or death occurred during the outbreak period; (2) infected individuals transmit the virus at a rate *λ* (transmission rate) and recover at a rate *γ* (recovery rate); and (3) the population was considered homogeneous, ignoring potential involvement of other species such as small ruminants (Van Den Driessche and Watmough 2002).

The following ordinary differential equations governed the system:

- dS/dt = −*λ* × S × I
- dI/dt = *λ* × S × I—*γ* × I
- dR/dt = *γ* × I

#### 2.6.1 Model Assumptions and Potential Sources of Bias

The assumption of homogeneous mixing within cattle herds was necessitated by the lack of detailed data on individual animal contacts, movement patterns, and interspecies interactions within the farms. However, FMD transmission in Ethiopia is known to be influenced by factors such as the presence of small ruminants, frequent milk container sharing, and occasional live animal movement between farms (Mohammed et al. 2022; Sulayeman et al. 2018). As these factors were not explicitly incorporated in the model, the estimated transmission rates (*λ*) and basic reproductive numbers (R_0_) should be interpreted as within‐cattle transmission potentials under minimal external contact influences, rather than representations of whole‐farm or multispecies transmission.

These assumptions were necessary due to limited animal movement and interspecies contact data during the outbreaks. Consequently, our estimates of *λ* and R_0_ should be interpreted as within‐cattle transmission potentials under controlled conditions and may underestimate true farm‐level transmission if between‐species or between‐farm contacts occur.

#### 2.6.2 Parameter Estimation

The transmission rate (*λ*) and recovery rate (*γ*) were estimated using the least squares method, fitting the model to the observed outbreak data (Cantó et al. 2017; Capaldi et al. 2012). Goodness‐of‐fit was evaluated using the sum of squared errors and root mean squared error. The basic reproduction number (R_0_), defined as the average number of secondary infections produced by one infected individual, was calculated as R_0_ = *λ*/*γ* (Diekmann 2025; Mehra et al. 2020; Van Den Driessche and Watmough 2002).

#### 2.6.3 Vaccine Effectiveness Adjustment

Based on field trial data from Ethiopia, the trivalent vaccine (serotypes O, A, SAT‐2) demonstrated 31% effectiveness (W. Jemberu et al. 2020). However, given the detection of SAT‐1 in mixed infections in our study, we adjusted the vaccine effectiveness downward to 26% to account for the lack of protection against this serotype. This adjusted value was used in critical vaccination coverage calculations.

#### 2.6.4 Critical Vaccination Coverage

The critical vaccination coverage (Vc) required to control FMD outbreaks was calculated using the formula: Vc = (1 − 1/R_0_)/Ve, where Ve represents the vaccine effectiveness, adjusted based on the effectiveness of the trivalent vaccine against the circulating serotypes (W. Jemberu et al. 2020).

### 2.7 Statistical Analysis

All data were analyzed using the R statistical software (R core team 2020). Descriptive statistics were used to summarize the distribution of FMDV detection and serotyping results. Transmission and recovery rates, as well as the basic reproductive number, were estimated for each farm.

## 3 Results

### 3.1 FMDV Detection and Serotyping

A total of 44 samples were collected from 11 dairy farms experiencing active FMD outbreaks. Out of these, 37 samples (84.1%) tested positive for FMDV using RT‐qPCR (Figure S1 and Table S1). Among the samples tested, 32 of 39 oral swabs (82.1%) and all five epithelial tissue samples tested positive for FMDV.

The results from the antigen capture ELISA assay revealed that 23 samples tested positive for FMDV. The detected serotypes included O, SAT‐2, and mixed serotypes involving SAT‐1 and SAT‐2, as well as O and SAT‐2. SAT‐2 was detected most frequently (8/23, 34.8%), followed by serotype O (4/23, 17.4%). Mixed infections involving O/SAT‐2 (5/23, 21.7%) and SAT‐1/SAT‐2 (3/23, 13.0%) were also observed. Furthermore, three samples could not be serotyped.

### 3.2 Transmission Dynamics

Using the SIR compartmental model, we estimated the within‐farm transmission dynamics of FMDV. The average daily transmission rate (*λ*) across all farms was calculated to be 0.82 (range: 0.48–1.14), and the average recovery rate (*γ*) was 0.32 (range: 0.12–0.90). The R_0_ estimated to be 3.7 (ranged: 1.4–6.8), indicating substantial heterogeneity in transmission intensity across dairy farms (Table 1). These findings suggest that FMDV transmission occurred rapidly within several farms, particularly in farms with high R_0_ values, where a single infected animal could potentially infect multiple susceptible animals over a short period.

**TABLE 1:** Transmission and recovery rates, along with basic reproductive number estimates for each farm.

ID No.	Transmission rate	Recovery rate	Basic reproductive rate	Predicted peak outbreak day	Observed peak outbreak days
Farm 1	1.13	0.47	2.4	5	5
Farm 2	0.84	0.56	1.4	4	5
Farm 3	0.87	0.47	1.8	2	—
Farm 4	0.99	0.31	3.1	6	5
Farm 5	0.67	0.12	5.9	8	11
Farm 6	0.91	0.14	6.2	6	6
Farm 7	0.58	0.13	4.3	9	12
Farm 8	0.48	0.12	3.7	11	11
Farm 9	0.50	0.15	3.3	9	11
Farm 10	0.86	0.12	6.8	6	7
Farm 11	1.14	0.90	1.5	7	5

Farms 5, 6, and 10 showed the highest R_0_ values (>5), indicating intense within‐farm transmission and a greater likelihood of rapid outbreak expansion. In contrast, Farms 2, 3, and 11 had relatively lower R_0_ estimates (<2), suggesting comparatively slower transmission dynamics and potentially better containment or lower effective contact rates among animals.

Model‐predicted outbreak peaks occurred between 2 and 11 days post‐first detection (Table 1). The highest number of clinically observed cases was recorded between days 3 and 12 across farms. In most farms, the predicted peak timings closely aligned with the observed outbreak peaks, indicating that the SIR model adequately captured the general progression of FMD outbreaks within the farms. However, discrepancies between model‐predicted and observed peak timings were noted in some farms and may reflect reporting delays, subclinical infections, delayed recognition of clinical signs, or variation in disease progression among animals.

**FIGURE 2:**
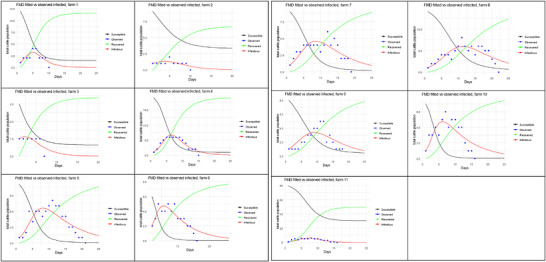
SIR model predictions of foot‐and‐mouth disease (FMD) outbreaks in dairy farms. The graph illustrates the dynamics of the outbreak across different farms, with the black, red, and green lines representing the model‐predicted numbers of susceptible, infected, and recovered individuals, respectively. The blue dots indicate the observed cases of infection recorded during the outbreak follow‐up, highlighting the alignment between the model predictions and actual infection data.

Utilizing the estimated transmission and recovery rates, the SIR model was applied to analyze the FMD outbreaks in each farm. In Figure 2, the black, red, and green lines represent the model‐predicted populations of susceptible, infected, and recovered individuals, respectively. The blue dots indicate the observed cases of infection recorded during the outbreak follow‐up. Overall, the observed data generally followed the predicted epidemic curves, supporting the usefulness of the model in describing the temporal dynamics of FMD outbreaks under field conditions.

### 3.3 Critical Vaccination Coverage

Considering the presence of mixed infections with SAT‐1 and SAT‐2, the effectiveness of the trivalent vaccine, initially observed at 31% from field trials, was adjusted to 26%. Based on this revised vaccine effectiveness, the critical vaccination coverage required to control the outbreak was calculated to be 280%. A critical vaccination coverage exceeding 100% indicates that vaccination with the current trivalent vaccine alone cannot achieve herd immunity under the observed transmission conditions.

## 4 Discussion

This study examines the transmission dynamics and serotypes of FMDV within dairy farms in Bishoftu, Ethiopia, where the disease poses a significant threat to livestock health and the local economy. The findings reveal a concerning 84.1% detection rate of FMDV among the tested samples, underscoring the endemic nature of the disease in the region. The predominant serotype identified was SAT‐2, followed by serotype O, along with mixed infections of SAT‐1 and SAT‐2, as well as O and SAT‐2. The detection of multiple serotypes and mixed infections highlights the complex epidemiology of FMDV in Ethiopian dairy systems and emphasizes the continuous risk of viral evolution and co‐circulation.

These results are consistent with previous studies documenting the evolving epidemiological landscape of FMDV serotypes in Ethiopia, highlighting the urgent need for ongoing surveillance to inform effective control measures. Among the five serotypes reported in the country, this study detected serotypes O, SAT‐1, and SAT‐2, with SAT‐2 emerging as the dominant serotype. This aligns with the findings of Ayelet et al. (2009), which also identified serotypes O, A, C, SAT‐1, and SAT‐2. Additionally, SAT‐2 has been reported as the predominant FMD serotype in central Ethiopia (Mohammed et al. 2022; Sulayeman et al. 2018).

The SIR model results demonstrated a significant potential for FMDV transmission within the farms, with an average R_0_ of 3.7. This suggests that each infected animal can infect more than three additional individuals, underscoring the epidemic risk associated with FMD. High transmission intensities suggest rapid amplification of FMD within confined dairy systems, especially where animal isolation is not practised immediately (Calkins and Scasta 2020). The variability in R_0_ values among farms (Table 1) indicates that farm‐specific factors, including herd management practices, biosecurity measures, and environmental conditions, significantly influence disease dynamics (Tadesse et al. 2019). For example, farms with higher R_0_ values may reflect inadequate biosecurity protocols, higher animal densities, or delayed responses to early signs of disease. Previous studies by Belayneh et al. (2020) and Tadesse et al. (2019) also reported reproductive numbers of 1.27 and 1.98, respectively; although these numbers are less than the current finding, they both indicate the epidemic nature of FMD in Ethiopia.

The effectiveness of the trivalent vaccine produced by the National Veterinary Institute, which includes serotypes O, A, and SAT‐2, was found to be only 31% in field trials (W. Jemberu et al. 2020). This level of effectiveness is notably low compared to the recommended threshold of over 75% protection (W. Jemberu et al. 2020), which raises serious concerns about the vaccine's ability to control FMD outbreaks effectively. Moreover, the vaccine confers immunity for only 6 months (Belsham 2020), necessitating frequent booster doses to maintain protection. Since the vaccine does not provide any efficacy against SAT‐1 infections, which were detected in this study, the reliance on the current trivalent vaccine is further compromised.

The estimated requirement for >100% vaccination coverage reflects the inadequacy of current vaccine effectiveness, demonstrating that herd immunity cannot be achieved through vaccination alone under current conditions. However, this estimate should be interpreted cautiously because vaccine effectiveness values were derived from previous outbreak‐specific field studies and were used primarily as contextual approximations for evaluating potential vaccination requirements. Vaccine performance in the field can vary substantially depending on antigenic matching between vaccine and circulating strains, vaccination schedules, cold‐chain maintenance, host immunity, and farm management conditions (Belsham 2020). In many endemic regions, reliance on vaccination alone is often insufficient to achieve disease control. Historical FMD outbreaks in various parts of the world, such as in the United Kingdom and South Africa, highlight the importance of a multifaceted approach that combines vaccination with additional strategies such as movement restrictions, isolation of infected animals, and enhanced biosecurity measures (Hayer et al. 2018; Keeling et al. 2003). The need for more potent vaccines that can confer broader protection against multiple serotypes is critical, as current vaccines often fail to account for the genetic diversity of FMDV circulating in the field (Grubman and Baxt 2004).

Several limitations should be acknowledged. First, our SIR model assumed homogeneous mixing and did not account for latent infection stages, between‐farm animal movements, or multispecies interactions, all of which may influence transmission dynamics. A Susceptible, Exposed, Infectious, Recovered (SEIR) framework incorporating an exposed (latent) compartment could provide a more biologically realistic representation of FMD transmission and should be considered in future studies with more detailed temporal outbreak data. Second, purposive sampling of clinically affected animals may have biased serotype detection toward more virulent strains. Third, vaccine effectiveness estimates were derived from previous field trials and may not fully reflect current field conditions in the study area.

## 5 Conclusion

This study demonstrated that FMD outbreaks in dairy farms in Bishoftu are characterized by high within‐farm transmission potential and predominance of the SAT‐2 serotype. The estimated R_0_ values indicate substantial transmission heterogeneity between farms, suggesting that management practices and biosecurity measures play an important role in outbreak dynamics. The detection of SAT‐1 alongside SAT‐2 and serotype O further highlights the complexity of FMD epidemiology in the area and raises concerns regarding the adequacy of the currently used vaccine formulation.

The findings suggest that vaccination alone is unlikely to achieve effective outbreak control under existing field conditions, particularly given the reported low vaccine effectiveness and limited serotype coverage. Therefore, effective FMD control in endemic dairy systems such as Bishoftu requires an integrated approach that combines vaccination with strengthened farm‐level biosecurity measures, including rapid isolation of infected animals, temporary movement restrictions during outbreaks, proper disinfection of shared equipment, and continuous monitoring of vaccine matching with circulating field strains. Strengthening surveillance and improving vaccine strain selection will be essential for reducing FMD transmission and minimizing economic losses in Ethiopia's dairy sector.

## Author Contributions

E.F. contributed to data collection, analysis, and manuscript writing. D.S., A.M., and A.A. performed laboratory analyses. J.P. contributed to conceptualization and supervision. F.T.W. contributed to conceptualization and supervision. H.N. contributed to conceptualization and supervision. S.L. contributed to conceptualization, supervision, data analysis, and manuscript writing.

## Funding

This study received support from the KEET joint project (project number: KE2019JOI016A101), a VLIR‐UOS project, Belgium. Moreover, laboratory facilities were supported by the Animal Health Institute (AHI) of Ethiopia. The funder had no role in the conception, design of the study, data collection, analysis, and interpretation of the data reported in this manuscript.

## Ethics Statement

Ethical approval for this study was granted by the animal research ethical review committee of the College of Veterinary Medicine and Agriculture of Addis Ababa University (reference number VM/ERC/03/12/2020). Animals were handled following the best veterinary care guidelines. Before conducting the research, animal owners were informed about the objectives and the benefits of the study, and they gave consent for their animal's inclusion in the study. The consent obtained from animal owners was verbal because owners are unable to write and read. Consent was taken in the presence of a third independent party and approved by the College of Veterinary Medicine and Agriculture of Addis Ababa University Ethics Committee.

## Conflicts of Interest

The authors declare no conflicts of interest.

## Supporting information




**Supporting File 1**: vms371054‐sup‐0001‐SuppMat.docx

## Data Availability

The data collected and used to support this study can be offered by the first author or the corresponding author upon request.
