# Supplementary material for: Serotype Characterization and Transmission Modelling of Foot‐and‐Mouth Disease in Dairy Farms, Bishoftu, Ethiopia
Source: Vet Med Sci. 2026 Jun 23;12(4):e71054. doi: 10.1002/vms3.71054 (PMC13288168; doi:10.1002/vms3.71054)
Supplement: Supplementary file 1 — Supporting File 1: vms371054‐sup‐0001‐SuppMat.docx [file VMS3-12-e71054-s001.docx]

Supplementary information


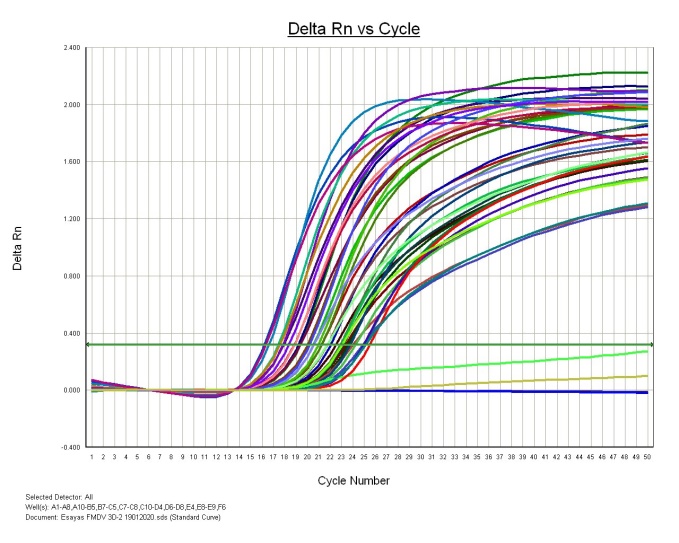


Supplementary figure 1: Real-time RT-PCR results for FMDV detection. The graph illustrates the amplification curves of the samples tested, with the threshold cycle (Ct) values indicated. Samples with Ct values below 32 are classified as positive for FMDV.

Supplementary table 1: Distribution of FMDV serotypes detected in sampled dairy farms in Bishoftu, showing farm scale, number of samples collected, number of positive cases, and serotypes identified during active outbreaks.

| ID No. | Number of animals in the farm | Farm scale | Number of sample collected | Number of samples positive (RT-qPCR) | Serotype detected |
| --- | --- | --- | --- | --- | --- |
| Farm 1 | 12 | medium | 5 | 5 | SAT-2, O & SAT-2 mixed |
| Farm 2 | 9 | small | 2 | 2 | O, SAT-2 |
| Farm 3 | 3 | small | 1 | 1 | - |
| Farm 4 | 18 | medium | 6 | 6 | O |
| Farm 5 | 11 | medium | 3 | 3 | - |
| Farm 6 | 7 | small | 7 | 5 | O, SAT-2, O & SAT-2 mixed |
| Farm 7 | 9 | small | 5 | 3 | SAT-2 |
| Farm 8 | 14 | medium | 3 | 3 | SAT-2, SAT-1 & SAT-2 mixed |
| Farm 9 | 8 | small | 5 | 2 | SAT-2 |
| Farm 10 | 7 | small | 5 | 5 | SAT-1 & SAT-2 mixed |
| Farm 11 | 80 | large | 2 | 2 | - |
